# Resistance to BET inhibitors in lung adenocarcinoma is mediated by casein kinase phosphorylation of BRD4

**DOI:** 10.1038/s41389-021-00316-z

**Published:** 2021-03-12

**Authors:** Jack Calder, Amy Nagelberg, Jennifer Luu, Daniel Lu, William W. Lockwood

**Affiliations:** 1 Integrative Oncology, British Columbia Cancer, Vancouver, BC V5Z 1L3 Canada; 2 Department of Pathology and Laboratory Medicine, University of British Columbia, Vancouver, BC V6T 2B5 Canada

**Keywords:** Non-small-cell lung cancer, Cancer therapeutic resistance

## Abstract

Targeting the epigenome to modulate gene expression programs driving cancer development has emerged as an exciting avenue for therapeutic intervention. Pharmacological inhibition of the bromodomain and extraterminal (BET) family of chromatin adapter proteins has proven effective in this regard, suppressing growth of diverse cancer types mainly through downregulation of the c-MYC oncogene, and its downstream transcriptional program. While initially effective, resistance to BET inhibitors (BETi) typically occurs through mechanisms that reactivate MYC expression. We have previously shown that lung adenocarcinoma (LAC) is inhibited by JQ1 through suppression of FOSL1, suggesting that the epigenetic landscape of tumor cells from different origins and differentiation states influences BETi response. Here, we assessed how these differences affect mechanisms of BETi resistance through the establishment of isogenic pairs of JQ1 sensitive and resistant LAC cell lines. We found that resistance to JQ1 in LAC occurs independent of FOSL1 while MYC levels remain unchanged between resistant cells and their JQ1-treated parental counterparts. Furthermore, while epithelial–mesenchymal transition (EMT) is observed upon resistance, TGF-β induced EMT did not confer resistance in JQ1 sensitive LAC lines, suggesting this is a consequence, rather than a driver of BETi resistance in our model systems. Importantly, siRNA knockdown demonstrated that JQ1 resistant cell lines are still dependent on BRD4 expression for survival and we found that phosphorylation of BRD4 is elevated in resistant LACs, identifying casein kinase 2 (CK2) as a candidate protein mediating this effect. Inhibition of CK2, as well as downstream transcriptional targets of phosphorylated BRD4—including AXL and activators of the PI3K pathway—synergize with JQ1 to inhibit BETi resistant LAC. Overall, this demonstrates that the mechanism of resistance to BETi varies depending on cancer type, with LAC cells developing JQ1 resistance independent of MYC regulation, and identifying CK2 phosphorylation of BRD4 as a potential target to overcome resistance in this cancer.

## Introduction

Epigenomic alterations are important drivers of tumorigenesis, modifying chromatin structure and the expression of key genes that influence cell phenotype. As such, inhibiting key components of the epigenetic machinery has become the focus of potential therapeutic strategies for many cancer types, as they have the potential to modulate gene expression programs associated with a diseased state^1^. The bromodomain and extraterminal (BET) family of proteins have become prime targets in this regard as they function as essential chromatin “readers” that recognize and bind to acetylated-lysine residues of histone tails to facilitate the activation of gene transcription^2–4^. BRD4 is the best characterized BET protein with a well-defined role in RNA polymerase II mediated transcription initiation and elongation^5^. BRD4 has been shown to occupy the enhancers of actively transcribed genes, particularly at large super-enhancer regions that are associated with the expression of transcription factors and other genes that drive cancer and tissue-specific cell development^6^. This role in mediating oncogenic potential combined with its recurrent genomic alteration in a subset of cancers, including NUT midline carcinoma, has increased interest in targeting BRD4 to inhibit diverse cancer types^7^.

BET inhibitors (BETis) have recently been developed that bind within the hydrophobic pocket of bromodomains preventing interaction with acetylated histones and transcription of target genes^8–10^. These inhibitors have proven to be effective in a wide range of cancers, including both hematological malignancies and solid tumors. For most of these cancers, sensitivity to BETis has been attributed to downstream suppression of MYC, a multifunctional transcription factor and key oncogenic driver of many tumor types^11–14^. These “MYC-dependent” cancers include those with activating MYC translocations such as acute myeloid leukemia (AML)^15^, B-cell acute lymphoblastic leukemia (B-ALL)^14^, Burkett’s lymphoma (BL)^13^, mixed-lineage leukemia (MLL)^16^, and multiple myeloma^12^, as well as neuroblastoma^17^, colorectal^5,18^, breast^19^, and pancreatic^20,21^ cancers. However, in contrast to its mechanism of action in these cancer types, we previously found that inhibition of lung adenocarcinoma (LAC) by the BET inhibitor JQ1 is c-MYC independent, with the oncogenic transcription factor FOSL1 functioning as the likely downstream target in this tumor type^22^. JQ1 treatment led to the downregulation of FOSL1 and AP-1 signaling in sensitive LAC cell lines, which were subsequently shown to be dependent on sustained FOSL1 expression for survival^22^. This effect was independent of driver gene mutation status as both EGFR and KRAS mutant LAC cell lines demonstrated differential sensitivity to JQ1 corresponding to FOSL1 suppression^22^. Thus, these findings suggest that the tissue origin and resulting epigenetic landscape of a cell is a crucial determinant in mediating the response and mechanism of action of BET protein inhibitors, an important consideration for the future development of therapies targeting epigenetic proteins in cancer cells. Since our initial study, osteosarcoma has also demonstrated MYC-independent sensitivity to BET inhibition, with FOSL1 as the likely downstream regulatory gene in this context as well^23^. More recently, MYC-independent BETi response has been reported in KRAS mutant pancreatic ductal adenocarcinoma, suggesting this may be a wide-spread phenomena^24^.

The efficacy of BETi treatment is being evaluated in many cancer types, with inhibitors currently at different stages of clinical trials^25^. However, as with most established cancer therapies, it is likely that both primary and acquired tumor resistance will limit effectiveness in patients^26^. One way to improve outcomes is through analysis of tumor mutational profiles and subsequent selection of patients with mutations or features characteristic of inhibitor sensitivity. While alterations in specific genes such as *LKB1*^27^, or activation of signaling pathways such as YAP/TAZ^28^ have been shown to impact BETi sensitivity, it remains unclear how prominent and generalizable these mechanisms are as the epigenetic landscape varies between patients. Furthermore, it is unknown whether these and other mechanisms mediating primary resistance play a role in acquired resistance.

Studies investigating acquired BETi resistance have been undertaken in many tumor types, mostly using the inhibitor JQ1^25^. However, in all instances, MYC-dependent cancers were used as model systems and resistance was universally conferred through reactivation of MYC expression, despite being mediated by different mechanisms, such as activation of Wnt signaling^15,29^, co-regulation by GLI2^20^, or phosphorylation of BRD4^19^. To date, acquired resistance to BETi treatment has never been studied in a MYC-independent cancer type, such as LAC, limiting the ability to combat other forms of BETi resistance in the clinical setting.

In this study, we investigated how LAC cells acquire resistance to JQ1 by establishing resistant isogenic cancer cell lines from initially sensitive cultures through dose escalation. Based on our previous work that demonstrated JQ1 sensitivity across different genetic subsets of LAC, we focused on EGFR and KRAS mutant cell lines as these represent the major driver genes in this type of lung cancer. LACs with mutations in EGFR typically develop resistance to kinase inhibitors—while those with KRAS mutations have no currently approved targeted therapy option—and new strategies to treat tumors driven by these oncogenes is required. We hypothesized that LAC lines would acquire resistance to BET inhibition through novel mechanisms, independent of MYC, and that this would identify novel genes/pathways that resistant tumors are dependent on for survival, opening up potential avenues for targeted combination therapy to improve the duration of BETi sensitivity in LAC driven by these oncogenes.

## Results

### Establishment of lung adenocarcinoma cell lines with acquired resistance to pharmacological inhibition of BET proteins

**Fig. 1:**
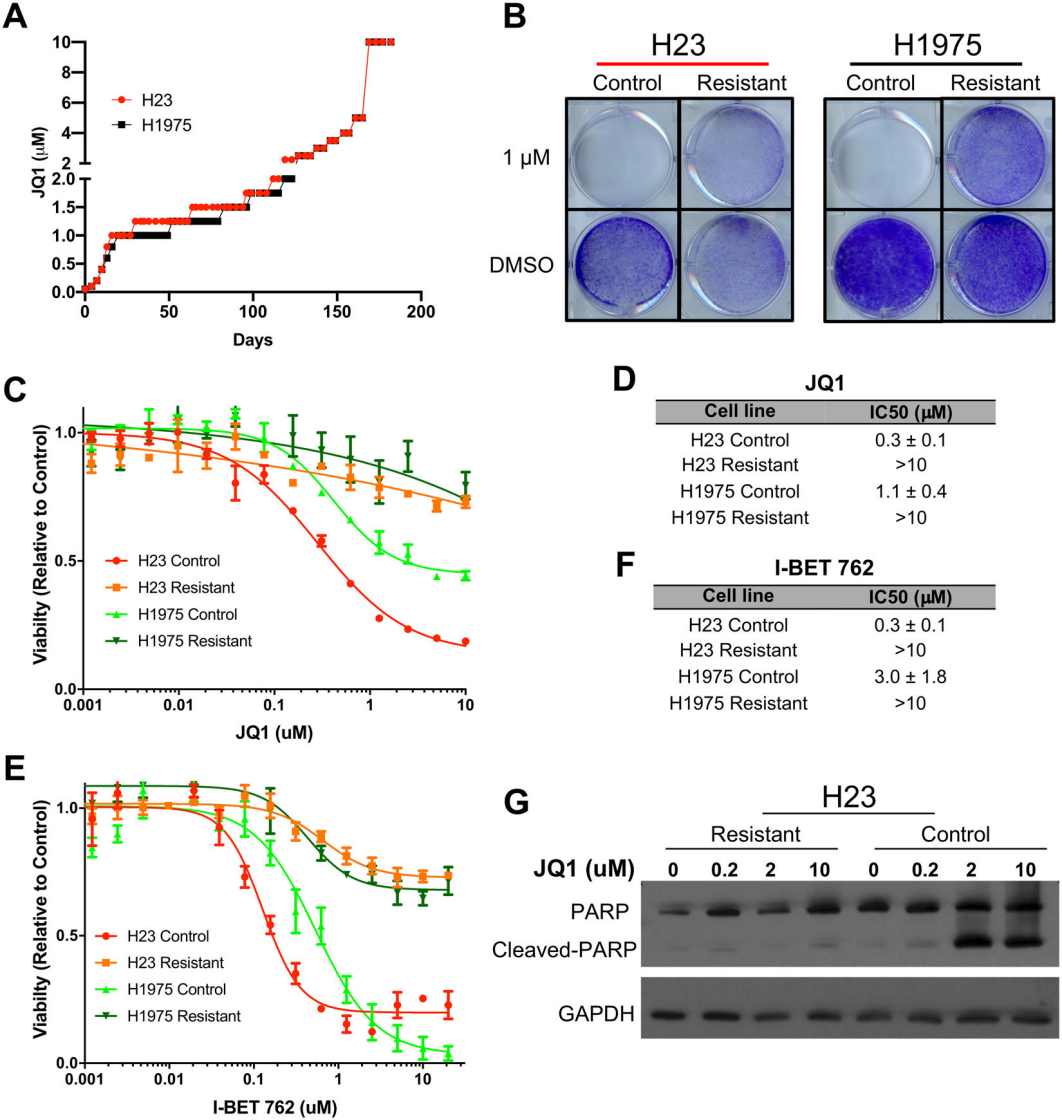
Establishment of JQ1 resistant lung adenocarcinoma cell lines. **A** Two JQ1 sensitive LAC lines, H23 and H1975, were cultured in increasing concentrations of JQ1 media, or 0.1% DMSO media as a control, for ~6 months until cells were able to continuously grow in 10 μM JQ1, where they were considered resistant. **B** Growth assays for H23 and H1975 resistant and control cell lines were performed with 1 μM JQ1 or 0.1% DMSO over 10 days followed by crystal violet staining to assess cell viability. **C** Dose response experiments were performed with JQ1 over 72 h with H23 and H1975 resistant and control cell lines. Each curve is of a representative experiment with four replicate values for each dose. **D** JQ1 IC_50_ values for each cell line are displayed from the average of four biological replicates ± standard error measure (SEM). **E** Representative dose response graph for I-BET762. **F** I-BET762 IC_50_ values from three biological replicates ± SEM. **G** Resistant and control H23 cells were cultured in escalating doses of JQ1 for 48 h. Western blots were performed to assess apoptosis through detection of cleaved PARP with GAPDH serving as a loading control.

To elucidate mechanisms of JQ1 resistance in LAC, two LAC cell lines we previously reported to be JQ1 sensitive, H23 and H1975—a KRAS^G12C^ mutant and an EGFR^L858R/T790M^ mutant line, respectively—were cultured with increasing concentrations of JQ1 over time until resistance was achieved. In total, cells were passaged over a 6-month period until the lines were able to grow in 10 μM JQ1, at which point they were considered resistant (Fig. 1A). In parallel, H23 and H1975 cells were also cultured in the same manner with vehicle only (media containing 0.1% DMSO) to control for effects associated with cell passaging (56 times for both lines). To confirm resistant lines were isogenic counterparts of their respective parental and control lines, short tandem repeat (STR) profiling cell authentication was conducted, with both H23 and H1975 resistant lines validated to match their respective control and parental counterparts (Supplementary Fig. 1).

JQ1 resistance was confirmed through clonogenic assays with both H23 and H1975 resistant lines demonstrating continued growth in 1 µM JQ1, while in contrast, control cell lines showed no viable cells after 10 days in the same conditions (Fig. 1B). Likewise, in dose response experiments, both resistant cell lines had JQ1 IC_50_ values greater than 10 µM, while the control lines had IC_50_ values of 0.3 μM (±0.1 μM) and 1.1 μM (±0.4 μM), for H23 and H1975, respectively (Fig. 1C, D). To further confirm that the cell lines were resistant to BET inhibition and not just JQ1 itself, another BETi, I-BET762 was used in dose response assays (Fig. 1E)^7^. As with JQ1, both resistant lines demonstrated IC_50_ values greater than 20 μM, the highest I-BET762 dose used, while H23 and H1975 control lines had IC_50_ values of 0.3 μM (±0.1 μM) and 3 μM (±1.8 μM), respectively (Fig. 1F). Furthermore, we saw evidence of apoptosis induction specifically in sensitive control cells, and not resistant cells, after JQ1 treatment as indicated by cleaved PARP (Fig. 1G). These results confirmed that we successfully established BETi resistant LAC lines for subsequent characterization.

### LAC lines acquire resistance to BET inhibitors in a MYC and FOSL1 independent manner

**Fig. 2:**
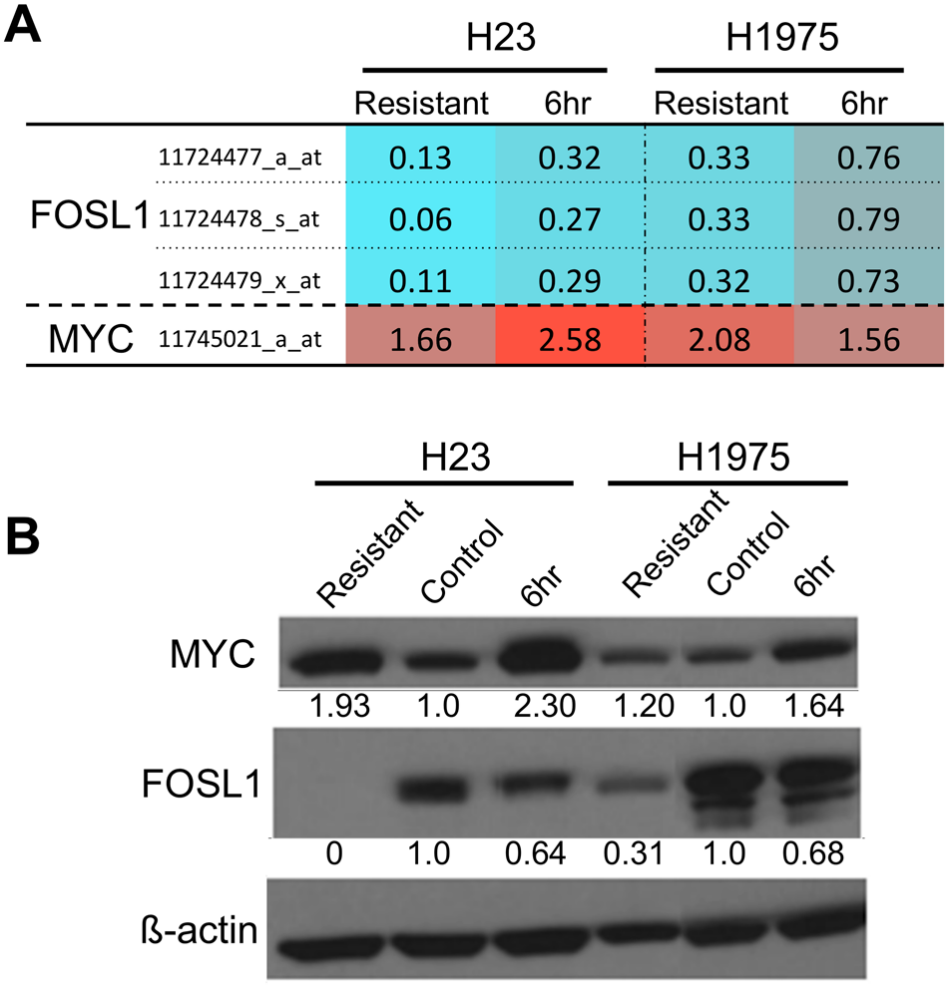
MYC and FOSL1 transcriptional activation are not involved in JQ1 resistance in LAC. **A** mRNA expression profiles for H23 and H1975 resistant, control and control cells treated with 10 μM JQ1 for 6 h were generated using Affymetrix arrays as described in the methods section. Resistant lines and 6 h 10 μM JQ1-treated lines were lysed while being cultured in 10 μM JQ1 while control lines were lysed, while being culture in regular media. Each condition was profiled in triplicate and the resistant and treated control conditions were compared against the control to determine the average fold change in expression. Probes mapping to FOSL1 and MYC are plotted, with the average fold change compared to control indicated. Color scale represents lower (blue) or higher (red) degree of expression compared to control. **B** Western blots for FOSL1 and MYC in the same cell lines and conditions as in (**A**). The numbers below each band indicate the relative change in protein levels compared to control, calculated using densitometry with beta-actin used to normalize loading. Both mRNA and protein indicate that MYC levels are higher upon JQ1 treatment (6 h), while FOSL1 levels both show initial decrease upon JQ1 treatment and further decrease in both resistant lines.

MYC and FOSL1 have previously been shown to be the two main effector targets that are downregulated due to BETi treatment in various cancers including LAC, with MYC reexpression being linked to multiple resistance mechanisms^15,19,20,22,29^. Therefore, it was hypothesized that reexpression of either of these proteins could confer resistance in our LAC model systems. To assess this, MYC and FOSL1 mRNA and protein levels were compared between resistant and control cell lines (Fig. 2). In addition, we also treated control lines with JQ1 for 6 h and assessed the levels of MYC and FOSL1 to determine the effects of acute JQ1 treatment in sensitive cell lines. MYC expression showed an increase of 1.66 and 2.08-fold in H23 and H1975 resistant lines, respectively, compared to their respective control lines, indicating that an increase in MYC expression could be a cause of resistance (Fig. 2A). However, MYC expression also increased 2.58 and 1.56-fold for H23 and H1975 control cells, respectively, upon 6 h JQ1 treatment, indicating that the changes in MYC expression are likely due to the presence of JQ1 and not a mechanism of resistance (Fig. 2A). This association was further confirmed by western blots as both resistant lines have higher levels of MYC protein as compared to control, but have less compared to the JQ1-treated control lines (Fig. 2B).

The upregulation of MYC in response to JQ1 treatment in LAC, as opposed to downregulation observed in other cancer types, is consistent with our previous report^22^. Likewise, we also validated that FOSL1 expression is decreased upon JQ1 treatment of control cell lines, further highlighting this transcription factor as the potential effector target of JQ1 response in LAC as we described previously (Fig. 2A). With this in mind, we predicted that FOSL1 may be re-expressed to drive JQ1 resistance in LAC lines; however, we found that FOSL1 was even further downregulated in both H23 and H1975 resistant cell lines compared to the JQ1-treated control cells (Fig. 2A). This was unexpected as previous studies had all shown that cancer cells acquired JQ1 resistance through reexpression of the initial effector target, in most cases, MYC^15,19,20,29^. However, this decrease in FOSL1 was confirmed at the protein level (Fig. 2B) with both resistant lines having lower levels of FOSL1 than their respective control lines. Therefore, we concluded that BET inhibitor resistance in H23 and H1975 LAC cells is independent of transcriptional activation of MYC or reactivation of FOSL1.

### Epithelial–mesenchymal transition (EMT) does not induce resistance to JQ1 in LAC

**Fig. 3:**
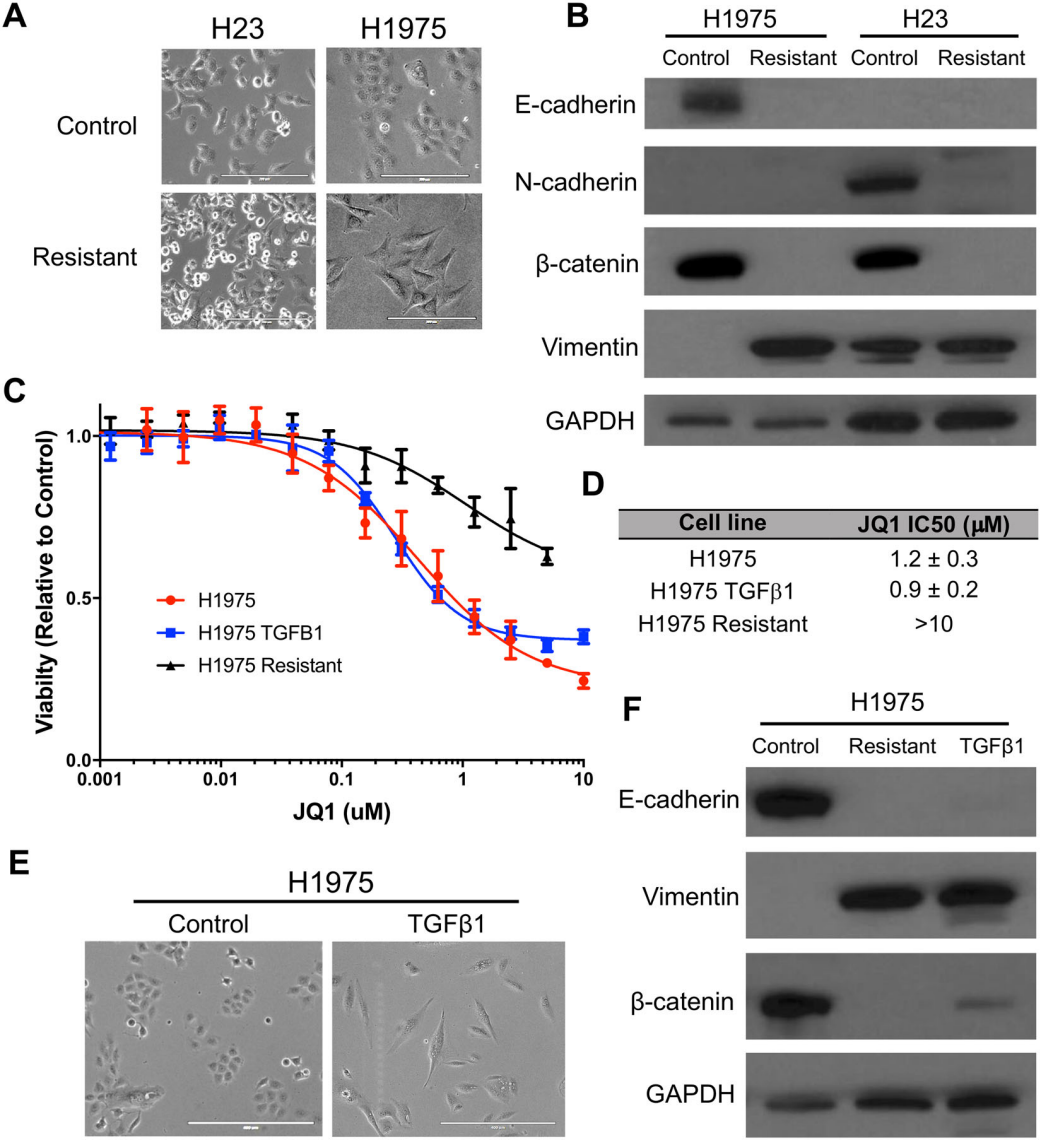
Induction of EMT is not sufficient to induce resistance to JQ1 in LAC. **A** Phase-contrast microscopy of H1975 indicates a mesenchymal morphology for the resistant line as compared to the epithelial morphology of the control line. These morphological differences are not seen in the H23 resistant line (scale bars = 200 μm). **B** Western blot analysis of EMT marker proteins parallels the morphological differences as the H1975 resistant line demonstrates downregulation of E-cadherin and β-catenin and upregulation of Vimentin, consistent with a shift towards a more mesenchymal phenotype. H23 shows no clear shift. GAPDH serves as a loading control. **C** H1975 parental lines were then treated with 10 ng/ml of TGFβ1 for 2 weeks to induce EMT and JQ1 sensitivity determined by 72 h dose response experiments. H1975 JQ1 resistant cells were also assayed for comparison. **D** JQ1 IC_50_ values representing the average of two experiments ± SEM. **E** Induction of EMT in H1975 cells by TGFβ1 treatment was assessed morphologically using phase-contrast microscopy (scale bars = 400 μm). **F** Western blot analysis of EMT marker proteins in H1975 cells treated with TGFβ1 confirm induction of a mesenchymal phenotype.

During the process of creating resistant lines we noticed that H1975 underwent a dramatic morphological change early in JQ1 dose escalation. This change in morphology constituted a shift from an epithelial phenotype (as still seen in the control line) to a more mesenchymal phenotype for the resistant line (Fig. 3A), suggesting the line underwent EMT. To confirm this, western blots for known EMT marker proteins were performed, confirming that the H1975 JQ1 resistant line had a mesenchymal phenotype, characterized by decreased levels of E-cadherin and β-catenin, and increased levels of vimentin, SLUG, Claudin-1, and ZEB1 (Fig. 3B, Supplementary Fig. 2). In addition, while H23 cells are already mesenchymal-like in terms of their marker expression, the H23 JQ1 resistant line also showed indications of transitioning to a more mesenchymal state, as SLUG increased and β-catenin and SNAIL levels decreased compared to control lines (Fig. 3B, Supplementary Fig. 2).

As this morphological transition was shown to be permanent (Supplementary Fig. 3) and EMT has previously been shown to be a driver of resistance to other inhibitors in LAC^30,31^, we next assessed whether EMT plays a role in BETi acquired resistance. To test if EMT could confer resistance to JQ1, H23 and H1975 parental cell lines were treated for 2 weeks with 10 ng/ml TGFβ1 to induce EMT^32^. JQ1 sensitivity was assessed by dose response experiments and induction of EMT was assessed morphologically and by western blot analysis, with the parental and resistant lines acting as benchmarks. For H1975, treatment with TGFβ1 showed induction of EMT both morphologically (Fig. 3E) and in protein marker expression, with decreased levels of E-cadherin and β-catenin and increased levels of vimentin paralleling the H1975 JQ1 resistant changes (Fig. 3F). However, TGFβ1 induced EMT was unable to confer JQ1 resistance, with IC_50_ values for these cell lines remaining similar to those of H1975 parental cells (0.9 μM ± 0.2 μM and 1.2 μM ± 0.3, respectively, Fig. 3C) and far from the IC_50_ of H1975 JQ1 resistant cells (>10 μM, Fig. 3D). TGFβ1 treatment of H23 had little effect on EMT marker expression aside from decreased levels of β-catenin and had no influence on JQ1 sensitivity (Supplementary Fig. 3). Therefore, these results suggest EMT is insufficient to induce BET inhibitor resistance in LAC.

### JQ1 resistant LAC cell lines are still dependent on BRD4 for survival

**Fig. 4:**
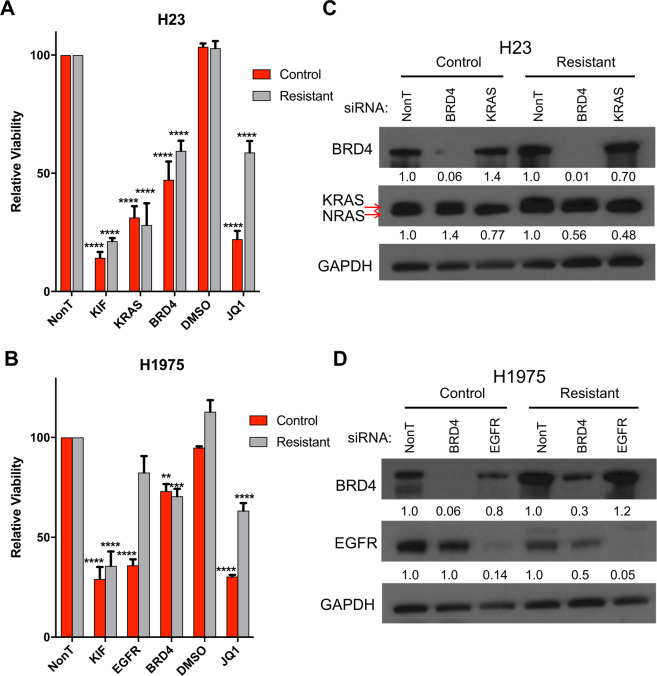
JQ1 resistant LAC cell lines are still dependent on BRD4 for survival. Relative viability following siRNA knockdown in H23 (**A**) and H1975 (**B**) JQ1 resistant and control lines. Cell lines were transfected with the indicated siRNAs and viability was assessed 96 h after transfection using Alamar Blue. Values are relative to the Non-target (NonT) siRNA control and represent the mean of triplicate experiments (error bars indicate SEM). KIF and KRAS/EGFR act as positive controls in the indicated cell lines. Asterisks denote level of statistical significance between the control and resistant line (*P < 0.05; **P < 0.01; ***P < 0.005; ****P < 0.001) using two-way ANOVA. **C**, **D** Western blot lysates were collected 72 h after transfection and analyzed, with GAPDH serving as a loading control. Relative change in protein levels (calculated by densitometry as in Fig. 2) for each cell line condition compared to NonT control are indicated below each band.

Next, we aimed to assess whether the JQ1 resistant cell lines were still dependent on BET proteins for survival. We inhibited BRD4—which is known to be the main BET protein targeted by JQ1 in LAC^22^—through RNAi mediated knockdown along with positive controls for cell death. This included Kinesin Family Member 11 (KIF11)—which is essential for mitosis in all cells^33^—as well as KRAS and EGFR, which are anticipated to selectively inhibit growth in LAC cell lines with these driver oncogenes. As expected, both KIF11 and EGFR/KRAS siRNA significantly (*p* value < 0.001, two-way ANOVA, *n* = 3) reduced the viability of both control H23 and H1975 cell lines, demonstrating successful knockdown, which was confirmed by Western blots (Fig. 4). These results were also seen in both resistant lines except for EGFR knockdown in the H1975 resistant line, which showed no significant change compared to the non-targeting siRNA (NonT) control. This indicates that the H1975 resistant line is no longer dependent on EGFR signaling, which may be related to EMT as previously reported^31^. As expected, BRD4 knockdown also significantly decreased viability of both control lines (H23 *p* value < 0.001, H1975 *p* value < 0.01, two-way ANOVA, *n* = 3). Surprisingly, however, BRD4 knockdown had a similar inhibitory effect on both JQ1 resistant lines, with no significant difference in viability observed compared to their respective JQ1 sensitive control cells (Fig. 4A, B). This effect was specific to BRD4 knockdown, as resistant cell lines in these assays were still insensitive to JQ1 treatment relative to control cells (Fig. 4A, B). Together, this suggests that LAC lines that have acquired resistance to JQ1 treatment are still dependent on BRD4, but likely not its bromodomain function, for survival.

### Increased phosphorylation of BRD4 by casein kinase 2 is associated with resistance to JQ1

**Fig. 5:**
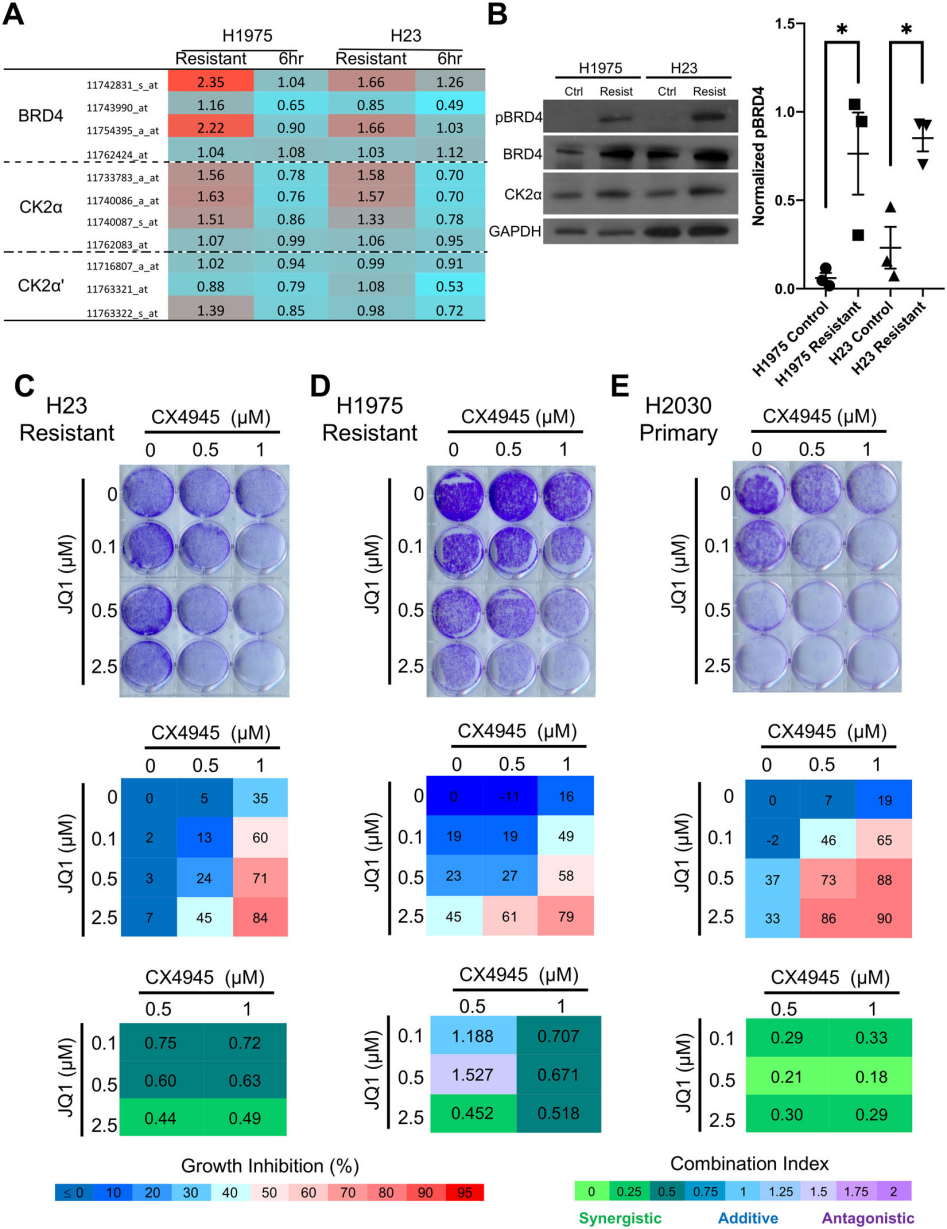
Phosphorylated BRD4 and the kinase CK2 are elevated in JQ1 resistant lines and are synergistic in inducing cell death when inhibited. **A** mRNA expression analysis shows increased expression of BRD4 and CK2α levels for resistant lines, but no change in CK2α′ levels. Values represent fold changes as compared to the control line for each specific cell line and condition. 11742831_s_at and 11754395_a_at are probes specific for the long isoform of BRD4. Color scale represents lower (blue) or higher (red) degree of expression compared to control. **B** Western blot analysis of pBRD4 and BRD4 levels, specific to the long isoform, as well as CK2α levels in JQ1 resistant and their control counterparts. Densitometry data for relative pBRD4 levels are plotted in scatter format for three independent western blots from independent cell lysates. GAPDH serves as a loading control and bars represent SEM (*P < 0.05, two-tailed, unpaired *T* test). **C**–**E** Combination growth assays for H23 (**C**) and H1975 (**D**) acquired resistant lines, and H2020 (**E**) primary resistant LAC cell line treated with the indicated combinations of JQ1 and CX-4945 for 10 days. Crystal violet images are representative of triplicate biological experiments. Cell viability was measured at the endpoint using Alamar Blue and the percent inhibition calculated compared to the no drug control, with the mean value of three biological replicates indicated. CompuSyn was used to calculate combination index (CI) scores for each drug combination with <0.75 indicating synergy, 0.75–1.25 indicating additive effects, and >1.25 indicating antagonism. Legends for percent growth inhibition and combination index values are shown at the bottom.

Previous studies have found that increased BRD4 levels are associated with primary resistance to BETi treatment^34^. To determine whether BRD4 overexpression could contribute to JQ1 resistance in LAC, we assessed BRD4 mRNA levels between JQ1 resistant and control cells using Affymetrix array profiling. We found that two probes, specific to the long isoform of BRD4 (1174283_s_at and 11754395_a_at, Fig. 5A) were upregulated in both H1975 and H23 JQ1 resistant lines compared to their respective controls. This was not observed in control cell lines treated with JQ1 for 6 h, suggesting that BRD4 upregulation is not a compensatory mechanism associated with JQ1 response. This BRD4 upregulation was also seen at the protein level, using an antibody that specifically detects the 200 kDa version of the protein, which represents the long isoform of the protein (Fig. 5B)^19^. Therefore, these results suggest that the resistant lines could be selectively increasing transcription of the long isoform of BRD4 to drive JQ1 resistance.

Phosphorylation of BRD4 (pBRD4) has also been shown to be associated with BETi resistance and BETi resistant cell dependency on BRD4 in a bromodomain-independent manner^19^. Upon assessment by western blot, we observed a significant increase in phosphorylation of the long isoform of BRD4 (pBRD4, as reported in^19^) in both JQ1 resistant LAC cell lines, with 12.7- and 3.7-fold more pBRD4 than controls for H1975 and H23, respectively (Fig. 5B). Casein kinase II (CK2) has previously been implicated as a kinase of BRD4, regulating its localization and stability^25^. To determine the role of CK2 in phosphorylating BRD4 in JQ1 resistant LAC cell lines, the two main genes that comprise the catalytic activity of the CK2 tetrameric kinase; casein kinase 2 alpha 1 (CK2α) and casein kinase 2 alpha prime (CK2α′)^35^, were assessed at the mRNA and protein level. While little difference was observed in CK2α′ expression, CK2α levels demonstrated an increase in both JQ1 resistant lines at the mRNA (Fig. 5A) and protein level (Fig. 5B). The parallel increase of both pBRD4 and CK2α levels suggest that CK2 may be associated with BRD4 phosphorylation in JQ1 resistant cell lines.

To examine whether CK2 plays a role in driving JQ1 resistance, the inhibitor CX-4945, which is known to inhibit CK2 activity and currently in clinical stages of development^36^, was used to treat H23 and H1975 resistant cell lines. Different concentrations of JQ1 and CX-4945 were used in combination and cell viability assessed using Alamar Blue followed by crystal violet staining at endpoint (10 days). Percent growth inhibition for each combination relative to control was calculated and CompuSyn, a program based on the Chou-Talalay principle, was used to calculate the combination index (CI) score for each drug combination^37^. We predicted that if CK2 is involved in JQ1 resistance, CX-4945 would synergize with JQ1 to inhibit the growth of resistant cell lines. Indeed, while JQ1 or CX-4945 had little effect on H23 and H1975 resistant cell lines alone, in combination they inhibited viability by ~80% at highest doses, a synergistic relationship as indicated by CI values < 0.75 (Fig. 5C, D; CI values between 0 and 0.75 indicate synergy, 0.75 and 1.25 indicate additive effects, and values >1.25 signify antagonistic effects). JQ1 and CX-4945 were also synergistic in the H23 and H1975 control cells, suggesting that combination treatment may improve initial response to BET inhibition (Supplementary Fig. 4). Therefore, as we have previously shown that there is a subset of LAC cell lines that display primary resistance to JQ1^22^, we next tested whether CK2 inhibition in these cell lines may also sensitize these lines to JQ1 treatment. To test this, the primary resistant line H2030 was selected, which was confirmed to be resistant to both JQ1 and I-BET762 at doses similar to acquired resistant LAC cells (>10 μM and >20 μM, respectively, Supplementary Fig. 5). Combination treatment of JQ1 and CX-4945 were performed as above, with the highest doses synergistically inhibiting H2030 cell viability by 90% (CI = 0.29, Fig. 5E). Together, these results indicate that CX-4945 and JQ1 act synergistically on BETi resistant LAC cells, providing further evidence that pBRD4, through CK2, may be a driver of resistance and that combination treatment may be a strategy to both improve initial response to JQ1 and prevent or delay the onset of resistance to single agent therapy.

### AXL and SPOCK1 are potential targets of pBRD4 and mediators of JQ1 resistance

**Fig. 6:**
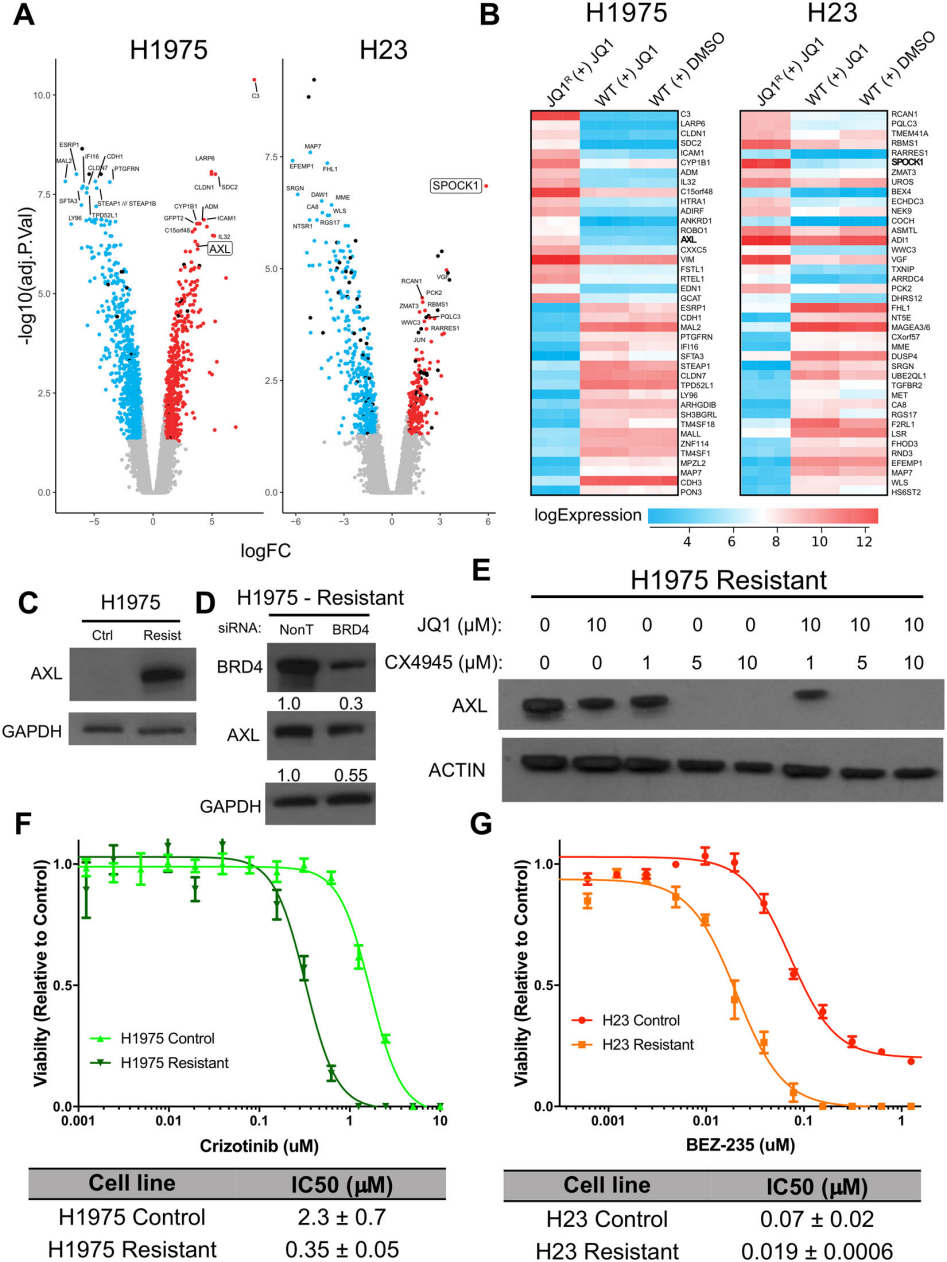
AXL and SPOCK1 are highly upregulated in JQ1 resistant lines and potential downstream effector targets of pBRD4. **A** Volcano plots for genes differentially expressed specifically in JQ1 resistant H23 (left) and H1975 (right) cells. Expression profiles were generated in triplicate as described in methods. Resistant cells and control cells treated with 10 μM JQ1 for 6 h were compared to control cells to identify significantly differentially expressed genes (>2-fold change, BH corrected *p* < 0.05, LIMMA) and the resulting lists compared to identify those deregulated only in the resistant lines. Genes significantly underexpressed (blue) or overexpressed (red) are indicated with selected gene names indicated. SPOCK1 and AXL are highlighted with a box around the gene name in H23 and H1975, respectively. **B** The top 20 up- and downregulated genes specific to the H1975 (left) and H23 (right) resistant lines. Expression values are plotted as a heatmap for the resistant line (JQ1^R^ + JQ1), control cells treated with 10 μM JQ1 for 6 h (WT + JQ1) and control cells in vehicle only (WT + DMSO). Values from triplicate experiments are shown for each condition. The color indicates higher (red) or lower (blue) expression (see legend bar). **C** Validation of AXL overexpression in H1975 resistant cells by immunoblot. Note, GAPDH is the same as in Fig. 3B. **D** siRNA mediated knockdown of BRD4 reduces expression of AXL in H1975 resistant cells. Protein levels are normalized to the NonT siRNA control condition. The blots for BRD4 and GAPDH are the same as in Fig. 4D. **E** Inhibition of CK2 with CX-4945 decreases the expression of AXL in H1975 resistant cells. Cells were treated with the indicated doses of CX-4945 and JQ1 for 6 h before lysis and blotting for the indicated proteins. Actin serves as a loading control. **F** Crizotinib dose response assays (72 h) in H1975 resistant and control cells. **G** BEZ-235 dose response assays (72 h) in H23 resistant and control cells. For (**F**, **G**), graphs are representative of a single experiment while IC_50_ values represent means from two biological replicates (±SEM).

In triple negative breast cancer (TNBC), hyperphosphorylated BRD4 has been shown to bind more strongly to MED1, facilitating bromodomain-independent chromatin localization^19^. However, the transcriptional targets of pBRD4 and their role in JQ1 resistance in LAC are unknown. To identify genes that may be targeted by pBRD4 to mediate acquired JQ1 resistance, genome-wide RNA expression profiling was performed using Affymetrix arrays. Resistant and control cells treated with JQ1 for 6 h were compared to control lines to identify differentially regulated genes in each group (Benjamini–Hochberg corrected *p* value < 0.05, fold change >2, LIMMA). Within each line, the resistant and treatment gene sets were compared to select for genes deregulated only in the resistant lines and not generally in response to JQ1 exposure. In total, 553 and 1494 genes were found to be differentially regulated specifically in H23 and H1975 JQ1 resistant cells, respectively (Fig. 6A).

We then assessed the top 20 up- and downregulated genes in each resistant cell line in hopes of identifying potential candidates that may be regulated by pBRD4 to drive resistance to JQ1 (Fig. 6B). Two of the top upregulated genes—AXL and SPOCK1 for H1975 and H23, respectively—were of interest as they are known to be involved in signaling pathways important to lung cancer development and resistance to targeted therapy (Fig. 6A,B). To confirm that AXL was upregulated in JQ1 resistant H1975 cells, immunoblots were performed that demonstrated a clear increase in protein expression in resistant cells, validating the gene expression data (Fig. 6C). To determine whether BRD4 regulates AXL expression in H1975 JQ1 resistant cells, we assessed AXL levels after siRNA mediated BRD4 suppression as described above. While BRD4 was not completely silenced, we observed ~50% reduction in AXL protein expression compared to the NonT siRNA control condition, suggesting BRD4 plays a role in driving AXL expression in resistant cells (Fig. 6D). Furthermore, inhibition of CK2 with CX-4945 completely reversed AXL expression in H1975 resistant cells, even in the absence of JQ1, indicating that AXL may be driven specifically by pBRD4 (Fig. 6E). To assess whether JQ1 resistant H1975 cells are dependent on AXL for survival, we performed dose response assays with the inhibitor Crizotinib, a known inhibitor of the kinase^38^. H1975 resistant cells had an IC_50_ of 0.35 μM ± 0.05, while the control line had an IC_50_ of 2.3 μM ± 0.7, confirming this premise (Fig. 6F). For H23 resistant cells, we performed a similar experiment to assess the potential dependency on SPOCK1, using the inhibitor BEZ-235 as it targets the phosphoinositide-3 Kinase (PI3K) pathway, the main pathway SPOCK1 is known to regulate^30,39^. Again, there was a clear shift to a lower IC_50_ in the resistant H23 cell line, suggesting increased dependency on PI3K/AKT signaling upon JQ1 resistance (Fig. 6G). Overall these results suggest that AXL and SPOCK1 may be upregulated by pBRD4 in the H1975 and H23 resistant lines, respectively, creating new dependencies that are targetable with inhibitors for their associated signaling pathways.

## Discussion

Targeting epigenetic factors for cancer therapy is a relatively new area of investigation that continues to evolve as we gain further understanding of the cellular mechanisms involved in epigenetic regulation. BET proteins, specifically BRD4, have demonstrated potential as drug targets in lung and other cancers leading to the initiation of a myriad of clinical trials^3,7,25^; however, mechanisms of response and resistance to these inhibitors are still poorly understood, especially across different cancer types. This may have consequences on clinical trial optimization and the development of strategies to circumvent resistance, delaying use of these drugs in patients that could benefit. Here, we describe the first investigation into mechanisms of BETi acquired resistance in LAC through the establishment of isogenic JQ1 sensitive and resistant cancer cell lines. We found that resistance to JQ1 is not driven by reactivation of FOSL1 or activation of MYC—two known targets of BRD4 inhibition—or by EMT, which was unable to induce resistance to JQ1. JQ1 resistant LAC cell lines are still dependent on BRD4 for survival, and pBRD4 is highly elevated in these lines, with a corresponding increase in the known BRD4 kinase CK2. Inhibition of CK2 synergizes with BETi to suppress growth of both primary and acquired JQ1 resistant LAC cell lines, offering a potential therapeutic strategy to increase response rates and duration in LAC patients. Lastly, we define two candidate genes potentially regulated by pBRD4 in JQ1 resistant LAC, AXL and SPOCK1, which cancers may become dependent on after adaptation to BETi treatment, offering additional avenues for therapeutic intervention.

While other studies have investigated mechanisms of JQ1 resistance, to our knowledge, this is the first to investigate acquired resistance in lung cancer. In addition, previous studies have focused on cancer types where MYC is the main effector target of BET inhibition^12–21,40^, and it was unknown whether mechanisms of resistance would be common across diverse cancers where JQ1 suppresses different targets. As the epigenetic landscape of cancer types vary significantly, it is important to understand how sustained BET protein inhibition leads to adaptation in these different contexts and try to identify potential commonalities that can be prioritized for therapeutic intervention. Our findings indicate that the phosphorylation of BRD4 may offer a prime target in this regard. Shu et al. have previously demonstrated that BRD4 is phosphorylated by CK2, and that increased levels of pBRD4 drive acquired resistance to BETi in TNBC^19^. This study also defined the long isoform of BRD4 as responsible for this effect and found that resistant tumors had reduced levels of the phosphatase PP2A, which opposes CK2 function on pBRD4. Reducing levels of PP2A increased pBRD4 and made TNBC cells less sensitive to JQ1 treatment, and the authors suggested that small molecule activators of PP2A enzymatic activity may offer a therapeutic approach to counteract BETi resistance^19^. While we did not observe changes in PP2A component expression (data not shown), we found that the catalytic component of CK2 (CK2α) is elevated in resistant LAC cell lines, creating sensitivity to the CK2 inhibitor CX-4945. This inhibitor is currently in clinical development^41–43^, offering a potential immediate strategy to improve response rates to BETi in ongoing clinical trials. Therefore, while the mechanisms regulating pBRD4 levels may differ across cancer types, targeting this key event may offer a ubiquitous approach for circumventing BETi resistance in these solid cancers.

While phosphorylation of BRD4 does not disrupt its binding to BETi such as JQ1, it is known to cause a switch in protein interactions^44^. In TNBC, it was demonstrated that pBRD4 binds more efficiently to MED1, allowing localization to acetylated lysines on histones through MED1 mediated chromatin binding and subsequent initiation of gene transcription in a bromodomain-independent manner^19^. The pBRD4-MED1 complex can then reactivate MYC expression, even in the presence of JQ1 mediated bromodomain suppression, leading to BETi resistance. This offers an alternative approach to reactivating anti-cancer targets of BRD4 than seen in other cancer types, such as in AML or leukemia, where β-catenin mediated transcription of MYC compensates for BRD4 to drive BETi resistance^26^. However, despite some commonality in targets, pBRD4-MED1 localizes to different chromatin regions than BRD4^19^, and is likely to cause the transcriptional activation of different genes. Indeed, in our LAC models of acquired JQ1 resistance, we did not see reactivation of the anti-cancer target of BETi in this cancer type, FOSL1. Instead, we observed broad activation of numerous genes that were transcriptionally silent in the parental, JQ1 sensitive LAC cells paralleled with the silencing of genes that were transcriptionally active in this JQ1 sensitive state. As pBRD4 is highly upregulated in the resistant cells, this suggests that there is a shift in chromatin localization to new target regions, leading to transcriptional rewiring that facilitates cancer cell survival. This change is epitomized by the main putative targets of pBRD4 in the H1975 and H23 JQ1 resistant cell lines, AXL and SPOCK1, respectively. AXL is not expressed in parental H1975 cells, but is highly elevated in resistant H1975 cells, and through CK2 inhibition we show that this is likely driven by pBRD4. Conversely, AXL is expressed in parental H23 cells, but suppressed in the JQ1 resistant counterpart (Supplementary Fig. 2, Supplementary Table 1). The opposite relationship is observed for SPOCK1 (Supplementary Table 1). Thus, pBRD4 and BRD4 can likely activate diverse and similar targets depending on the specific cellular context and the presence of epigenetic co-factors, and greater work is needed to explore these relationships and their role in driving genes responsible for mediating BETi resistance.

Aside from CK2 inhibition, the identification of AXL and SPOCK1 revealed additional dependencies in JQ1 resistant LAC that may be pharmacologically exploited. We found that the clinically approved inhibitor crizotinib—which inhibits AXL^38,45^ in addition to main targets ALK and ROS1—is effective in suppressing the growth of JQ1 resistant H1975 cells that display AXL overexpression. AXL has previously been implicated in EGFR inhibitor resistance in H1975 cells, suggesting that this acts as a bypass pathway to compensate for the baseline signaling and driving survival in this cell line^46^. Furthermore, AXL is known to mediate cancer cell phenotypic plasticity and EMT^47,48^, suggesting that upregulation may be related to the morphological shift of resistant H1975 cells to a mesenchymal-like state (Fig. 3A, B). Importantly, knockout of AXL was recently identified in a large scale CRISPR screen to be synthetically lethal in the context of JQ1 resistant TNBC, further implicating this receptor as a potential target to circumvent BETi resistance^49^. Lastly, AXL is known to activate YAP signaling in EGFR mutant LAC^50^, which has recently been associated with JQ1 sensitivity and resistance in both breast and lung cancer^28,51^. Interestingly, gene set enrichment analysis revealed YAP1 activation as the second most enriched pathway associated with JQ1 resistance in H1975 cells (Supplementary Table 2) coinciding with AXL upregulation. Together, this suggest that AXL may be a main downstream transcriptional target of pBRD4 that drives survival and EMT to circumvent the effects of JQ1 treatment.

SPOCK1 has recently been implicated in numerous aspects of tumorigenesis including the positive regulation of cell proliferation, invasion, migration and survival^52^. The main signaling role for SPOCK1 is in regulation of the PI3K/AKT pathway^30,39^, and we found that treatment of SPOCK1 overexpressing H23 JQ1 resistant cells with the PI3K inhibitor BEZ-235 (dactolisib) reduced the viability of these cells compared to JQ1 sensitive controls. Interestingly, in prostate cancer, increased PI3K/AKT signaling has been implicated in primary resistance to BETi, which is driven through mutations in the E3 ubiquitin ligase substrate-binding adaptor speckle-type POZ protein (SPOP) that increased BRD4 protein levels due to impaired SPOP-mediated degradation^53^. Whether or not pBRD4 is increased as a result of SPOP mutation, and if SPOCK1 induction mediates the increased PI3K/AKT signaling in prostate cancer is unknown. However, our findings suggest that AXL and the PI3K/AKT pathways may provide additional combination strategies in conjunction with BETi to improve response rates in lung and other cancers.

Together this work furthers our understanding of how LAC lines respond to BETi treatment and identifies specific targets and pathways essential for resistance. BETi have shown modest activity in lung cancer. We propose a potential strategy for combination-based therapy that employs CK2 inhibitors to potentiate the effects of BET inhibition in lung cancer cells by suppressing the phosphorylation of BRD4. This approach may both increase the initial activity and response rate of LAC to JQ1, as well as increase the duration of response by preventing pBRD4 driven transcriptional changes that mediate acquired resistance. Combining this strategy with other therapeutic options for both EGFR (tyrosine kinase inhibitors) and KRAS (immunotherapy, KRAS-^G12C^ inhibitors) mutant LACs may also provide synergetic effects and should be the focus of future preclinical and translational studies. Lastly, the role of pBRD4 in driving BETi resistance in distinct lung cancer subtypes, in particular BRD4-fusion NUT midline carcinomas, as well as other cancers types with MYC-independent JQ1 sensitivity and driver gene mutations common to LAC—for example KRAS mutant pancreatic cancer^24^—warrants investigation and should be further explored.

## Materials and methods

### Cell lines and culture conditions

NCI-H23 (H23), NCI-H1975 (H1975), and NCI-H2030 (H2030) LAC lines were purchased from the American Type Culture Collection (ATCC) and cultured in RPMI-1640 medium (Gibco, cat. #11875119) supplemented with 5% (vol/vol) FBS (Sigma), 1% penicillin–streptomycin solution (Gibco), and 1% Glutamax (Gibco) (standard growth media). Cultured cells were incubated in a humidified incubator at 37 °C with 5% (vol/vol) CO_2_ and 95% (vol/vol) air.

### Establishing resistant lines

JQ1 resistant lines were generated by treating parental H23 and H1975 cells with increasing concentrations of JQ1 over a period of 6 months until cells could grow in 10 μM JQ1. In parallel, cells were cultured in standard growth media plus 0.1% (vol/vol) DMSO as a control. Resistant lines were maintained in 10 μM JQ1 media and the control lines in 0.1% DMSO media. Both resistant and control lines were tested for mycoplasma contamination and authenticated by STR profiling through Genetica.

### Dose response experiments and long-term growth assays

Dose response experiments entailed 21-point doubling dilutions (in DMSO) starting at 10 μM for JQ1, Crizotinib, and BEZ-235 and 20 μM for I-BET762. 10 μL of each dilution was added in quadruplicate to wells of a 96-well plate (Falcon). 10 μL of 100 μM of Etoposide (Selleckchem, cat. # S1225) was also added in quadruplicate to four wells of the 96-well plates. Cells were then added (in 90 μL complete growth media) at 3000–8000 cells per well, based on growth rate to ensure optimal final densities, with final drug concentrations having a 0.1% DMSO concentration (vol/vol). Plates were incubated (at previously described conditions) for 72 h at which time 10 μL (10% of total volume) of Alamar Blue cell viability reagent (Invitrogen, cat. # Dal1100) was added and fluorescence measured using a BioTEk Cytation 3 imaging reader (Excitation: 540 nm and Emission: 590 nm). Results were obtained using BioTek Gen5 software version 2.06.10. IC_50_ values were generated using GraphPad Prism 7 software and are presented as the mean ± SEM (Standard error of the measurement) of between 2 and 4 biological replicates, as indicated.

For long-term cell growth assays cells, 20,000 cells were seeded into six-well plates (Falcon) for H1975 control and resistant lines; 25,000 for H23 control line; and 50,000 for H23 resistant line. Cells were seeded in complete growth media with either 1 μM or 10 μM JQ1, or equivalent vehicle (0.1% DMSO) as a control, with fresh media being exchanged every 2–3 days. On day 10, media was aspirated and cells stained with Crystal violet (CV) staining solution (Sigma) (50% CV, 25% methanol, 25% PBS) for 30 min before rinsing plates of excess CV solution and imaging the following day. Experiments were repeated at least twice.

### RNA interference (RNAi) experiments using siRNAs

Cells were seeded into antibiotic-free standard growth media in six-well plates, with seeding densities ranging from 200,000 for H1975 control and resistant; 250,000 for H23 control; and 350,000 cells per well for H23 resistant. Wells were checked the following day (24 h) for ~70–80% confluency before continuing. Cells were transfected with 10 μL of 10 μM ON-TARGETplus SMARTpool siRNAs (Dharmacon), specific to the gene of interest or a non-targeting control (NonT), using DharmaFECT 1 transfection reagent (Dharmacon) according to the manufacturer’s instructions. 16–18 h after transfection cells were trypsinized and counted, and then re-seeded in 96-well plates at 2000 (H23 control, H1975 control and H1975 resistant) and 4000 (H23 resistant) cells per well, in quadruplicate for each condition. Cells were also re-seeded into 6 well plates for an additional 48 h and then lysed using 100 μL radioimmunoprecipitation assay (RIPA) lysis buffer (Pierce Biotech) containing Halt Protease and Phosphatase Inhibitor Mixture (Thermo Scientific). Cell growth was analysed 96 h after re-seeding into 96-well plates using Alamar Blue as described above and fluorescence measured as previously described. Viability for each siRNA was averaged between the four replicates and then compared to the NonT control (set at 100% viability) to calculate relative viability. For each line, resistant and control values for each specific siRNA were compared using the two-way ANOVA (Analysis of variance) test in the Prism 7 software package (GraphPad), with significance being denoted as ns = not significant, *P < 0.05; **P < 0.01; ***P < 0.005; ****P < 0.001. The two-way ANOVA test was also used to determine significance of siRNA to NonT within each control and resistant line. Each experiment was performed in triplicate.

### CX-4945 and JQ1 combination treatment

For combination growth assays, cells were seeded into six-well plates (Falcon) at 10,000–15,000 cells/well (H2030, H23 control, H1975 control, and H1975 resistant) and 40,000–50,000 cells/well (H23 resistant) for 10-day treatment. Media was changed with fresh media with inhibitor every 2–3 days. Cells were seeded in regular culture media and then the media changed the following day with media containing inhibitors or vehicle control, which was designated the 0 time-point/day. After 10 days, cell viability was measured by addition of 200 μL Alamar Blue to each well (2 mL of media, 10%) and then quadruplicate 100 μL samples extracted and put into a 96-well plate and fluorescence measured as previously described. After extracting the 100 μL samples the rest of the media was aspirated and cells stained with CV staining solution (Sigma) (50% CV, 25% methanol, and 25% PBS) as above. All experiments were performed in biological triplicate.

### Quantitative analysis of combinational treatment

For each experiment, the four technical replicates were averaged and then compared to the control well (0 JQ1, 0 CX-4945), which was set as 100% viability. These values for each combination were then averaged from the three biological replicate experiments to give the percent growth inhibition for each combination of inhibitors. Drug synergism was analyzed from these average values using CompuSyn software (www.compusyn.com), which is based on the Median-Effect Principle (Chou and the Combination Index-Isobologram Theorem (Chou-Talalay)^37^. Following the instructions of the software, drug combinations at nonconstant ratios were used, with any values >0.99 being changed to 0.99, to allow the software to calculate Combination Index (CI) values, where CI < 0.75 indicates synergism; CI between 0.75 and 1.25 indicates additive effects, and CI > 1.25 indicates antagonism.

### Gene expression profiling and data analysis

In triplicate, RNA was extracted using the RNeasy Mini Kit (Qiagen) from H23 and H1975 resistant lines (growing in 10 μM JQ1 media), control lines (growing in 0.1% DMSO media), and control lines treated with 10 μM JQ1 for 6 h, all from independent treatments in six-well plates. Samples were sent to The Center for Applied Genomics (TCAG, Toronto, Canada) where sample quality, sample labeling, array hybridization, and data acquisition were performed, with the Affymetrix Human PrimeView Array being used as previously described^22^. Data has been uploaded to the Gene Expression Omnibus (GEO, Accession Number GSE164813).

Data was normalized by Robust Multiarray Analysis^54^ as previously described^55^. Therefore, for each probe, triplicate expression values (in log_2_ scale) for resistant, control, and 6 h treated cells were obtained. The limma R package available through Bioconductor was used to determine significant differentially expressed (DE) genes^56,57^. A Benjamini–Hochberg corrected *p* value < 0.05 together with fold change greater than 2 were considered significant for all analyses. Resistant cell lines and 6 h JQ1-treated WT lines were independently analyzed versus the untreated controls. Genes that were significant within the treated controls were then excluded from further analysis within the resistant cell lines. Data were plotted using various packages available through R^58–60^. Gene set variation analysis was used in conjunction with the MSigDB oncogenic signature gene set collection (C6) to identify pathways differentially regulated between JQ1 resistant and control counterparts, as well as JQ1-treated and control cells^61^. Gene signatures with a Benjamini–Hochberg corrected *p* value < 0.05 were considered significant, and those found in resistant cells were filtered on gene sets significant in their respective JQ1-treated control cells to determine those potentially mediating resistance.

### Protein extraction and western blot analysis

Cells were grown in either six-well or 6 cm plates and were lysed with 50–200 μL of RIPA lysis buffer (Pierce Biotech) containing Halt Protease and Phosphatase Inhibitor Mixture (Thermo Scientific), after washing with ice cold phosphate-buffer saline (PBS). Cells were harvested by scraping and placed at −80 degrees at least overnight. Cell lysates were sonicated, cleared of cell debris through centrifugation at 18,000 × *g* for 15 min, and then protein concentration determined using the Pierce BCA Protein Assay kit (Thermo Scientific) according to the manufacturer’s instructions, with absorbance being measured at 562 nm using a plate reader as previously described. 25 μg of protein was used for each sample and denatured by boiling at 100 °C for 10 min in 4× Laemmli sample buffer (BioRad) with 1:10 addition of 2-Mercaptoiethanol (Thermo Fisher). Samples were loaded on Novex 4–12% Bis Tris Gels (NuPage) and electrophoresed at 150 V for 2 h, then transferred to polyvinylidene fluoride (PVDF) membranes (Millipore) at 110 V for 1 h 10 min. Membranes were blocked at room temperature for 1–2 h with either 5% (wt/vol) non-fat dry milk or 5% (wt/vol) bovine serum albumin (BSA) (Sigma), made up in TBS-T (1× Tris-buffered saline, 0.1% (vol/vol) Tween-20) (TBS BioRad), followed by immunoblotting with primary antibodies (described below) overnight at 4 °C with shaking. Membranes were incubated with the appropriate horseradish peroxidase (HRP) conjugated secondary antibodies (donkey anti-rabbit (sc-2313) and donkey anti-mouse (sc-2314) from Santa Cruz) at 1:2000-1:10,000 dilutions for 1.5 h at room temperature and subsequent detection using SuperSignal West Pico Chemiluminescent Substrate (34087) or SuperSignal West Femto Chemiluminescent Substrate (PI34095) (Pierce Biotech).

### Antibodies, inhibitors, and other reagents

All antibodies used for western blot analysis were 1:1000 dilution except for CK2α (1:500) and GAP (1:2000) and are as follows: From Cell Signaling Technology (CST); BRD4 (13,440), AXL (8661S), E-cadherin (3195), N-cadherin (13,116), β-catenin (8480), Vimentin (5741), EGFR (2232S), Ras (8955S), EGFR (L858R mutant specific) (3197), MYC (5605), and FOSL1 (5281). From abcam; CK2α (ab137788), and from Santa Cruz; GAP (sc-47724). Lastly the pBRD4 antibody was a gift from C. M. Chiang with specificities as previously described^44^ and used to detect pBRD4 as described in^19^. (+)-JQ1 (active enantiomer) (cat. # 4499) was purchased from TOCRIS. I-BET762 (cat. # 10676) was purchased from Caymen Chemical. Crizotinib (cat. # S1068) and CX-4945 (also known as Silmitasertib) (cat# S2248) were purchased from Selleck Chemical, and BEZ235 (cat. #CT-BEZ) was purchased from ChemieTEK. All drugs were suspended in DMSO. Recombinant human TGF-beta 1 (TGFβ1) protein was purchased from R & D Systems (cat. # 240-B-002) and resuspended in 4 mM HCl containing 1 mg/mL BSA to a final concentration of 10 μg/mL. This was then added to regular cultured media at 1:1000 to give a final concentration of 10 ng/mL.

## Supplementary information

Supplemental Figures 1-5

Supplemental Table 1

Supplemental Table 2
