# Supplementary material for: Resistance to BET inhibitors in lung adenocarcinoma is mediated by casein kinase phosphorylation of BRD4
Source: Oncogenesis. 2021 Mar 12;10(3):27. doi: 10.1038/s41389-021-00316-z (PMC7955060; doi:10.1038/s41389-021-00316-z)
Supplement: Supplementary file 1 — Supplemental Figures 1-5 [file 41389_2021_316_MOESM1_ESM.pptx]

## Slide 1
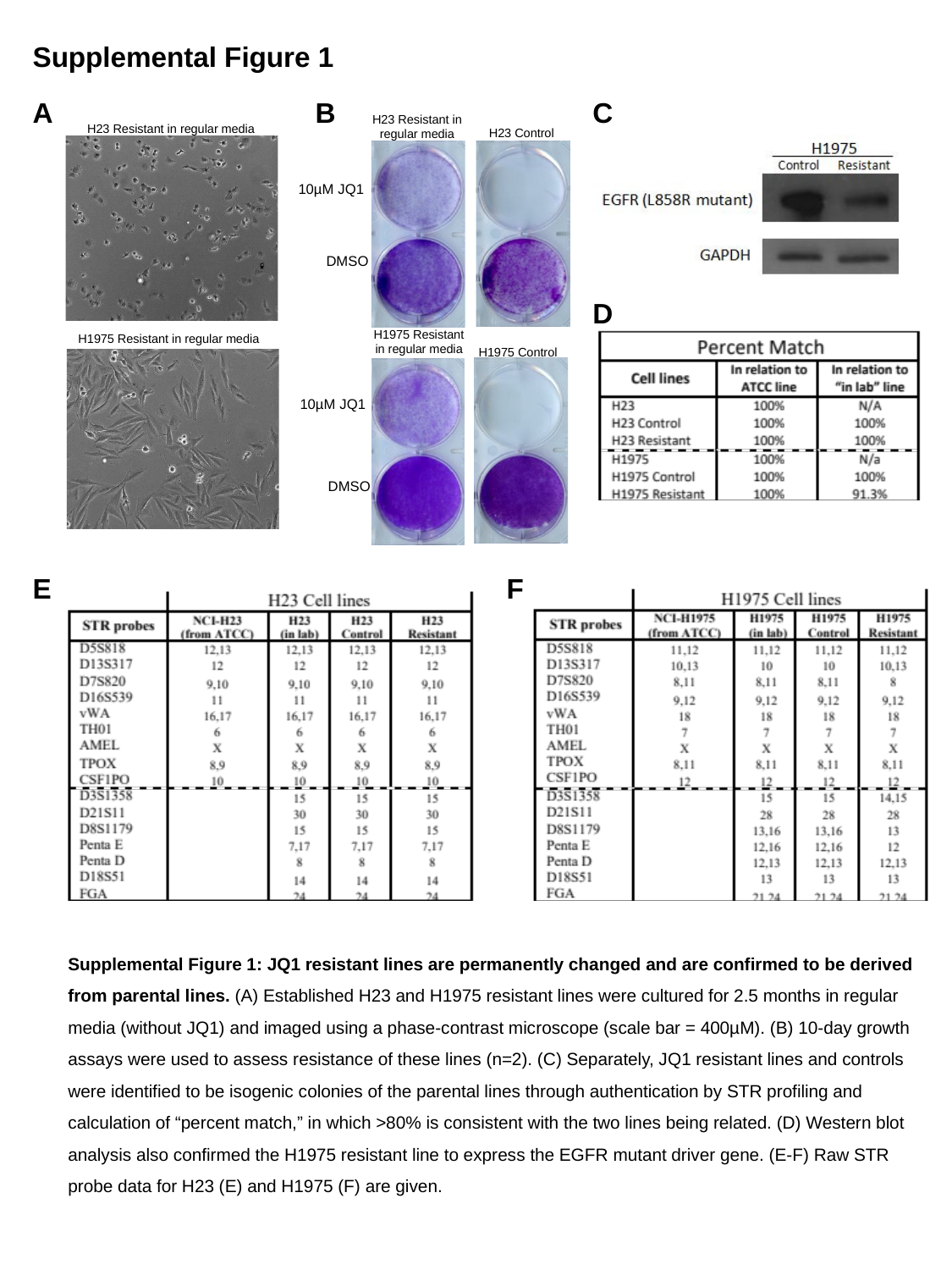

Supplemental Figure 1
A
B
C
H23 Resistant in regular media
H23 Control
10µM JQ1
 DMSO
H23 Resistant in regular media
D
H1975 Resistant in regular media
H1975 Control
10µM JQ1
 DMSO
H1975 Resistant in regular media
E
F
Supplemental Figure 1: JQ1 resistant lines are permanently changed and are confirmed to be derived from parental lines. (A) Established H23 and H1975 resistant lines were cultured for 2.5 months in regular media (without JQ1) and imaged using a phase-contrast microscope (scale bar = 400µM). (B) 10-day growth assays were used to assess resistance of these lines (n=2). (C) Separately, JQ1 resistant lines and controls were identified to be isogenic colonies of the parental lines through authentication by STR profiling and calculation of “percent match,” in which >80% is consistent with the two lines being related. (D) Western blot analysis also confirmed the H1975 resistant line to express the EGFR mutant driver gene. (E-F) Raw STR probe data for H23 (E) and H1975 (F) are given.

## Slide 2
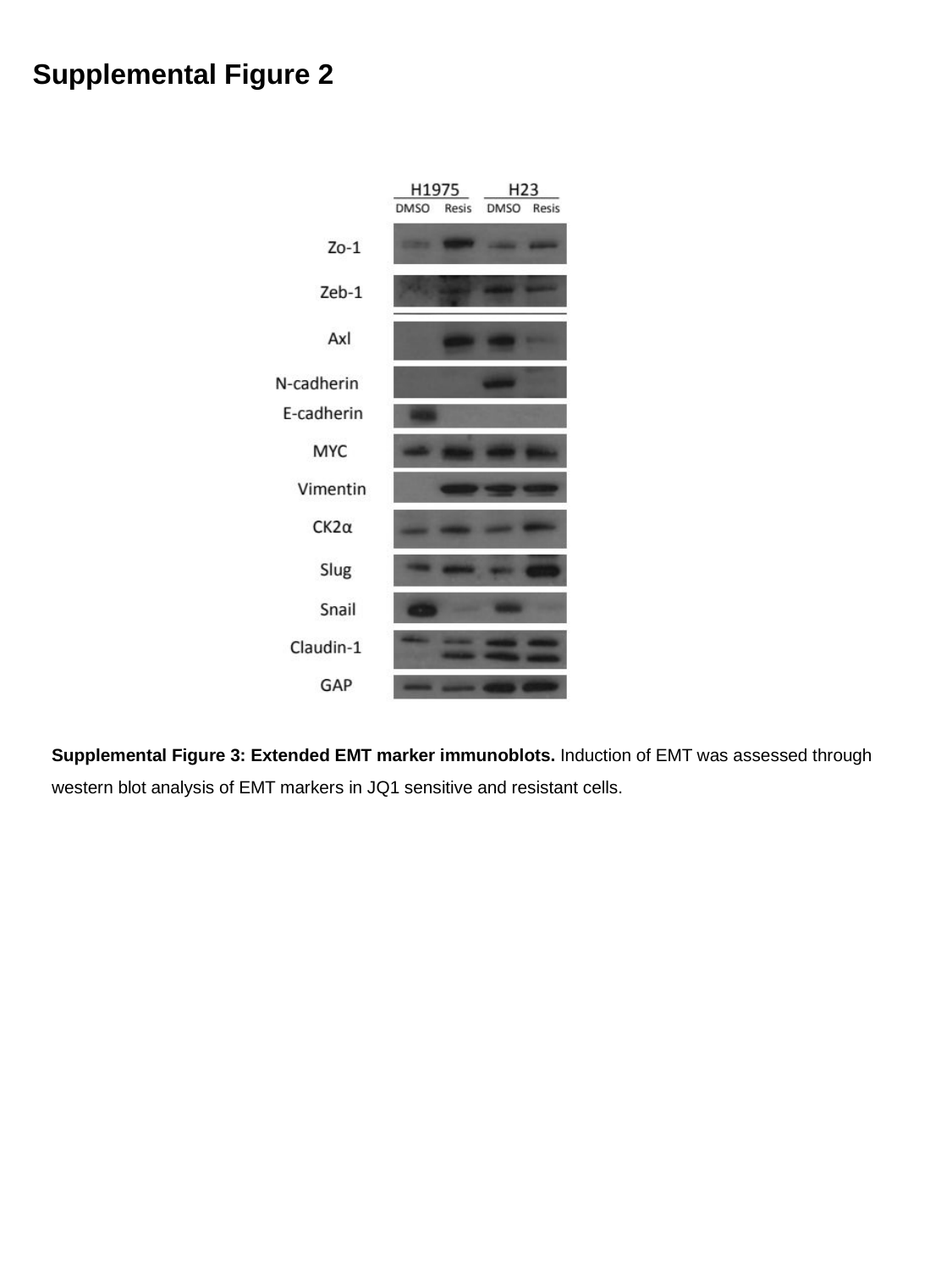

Supplemental Figure 2
Supplemental Figure 3: Extended EMT marker immunoblots. Induction of EMT was assessed through western blot analysis of EMT markers in JQ1 sensitive and resistant cells.

## Slide 3
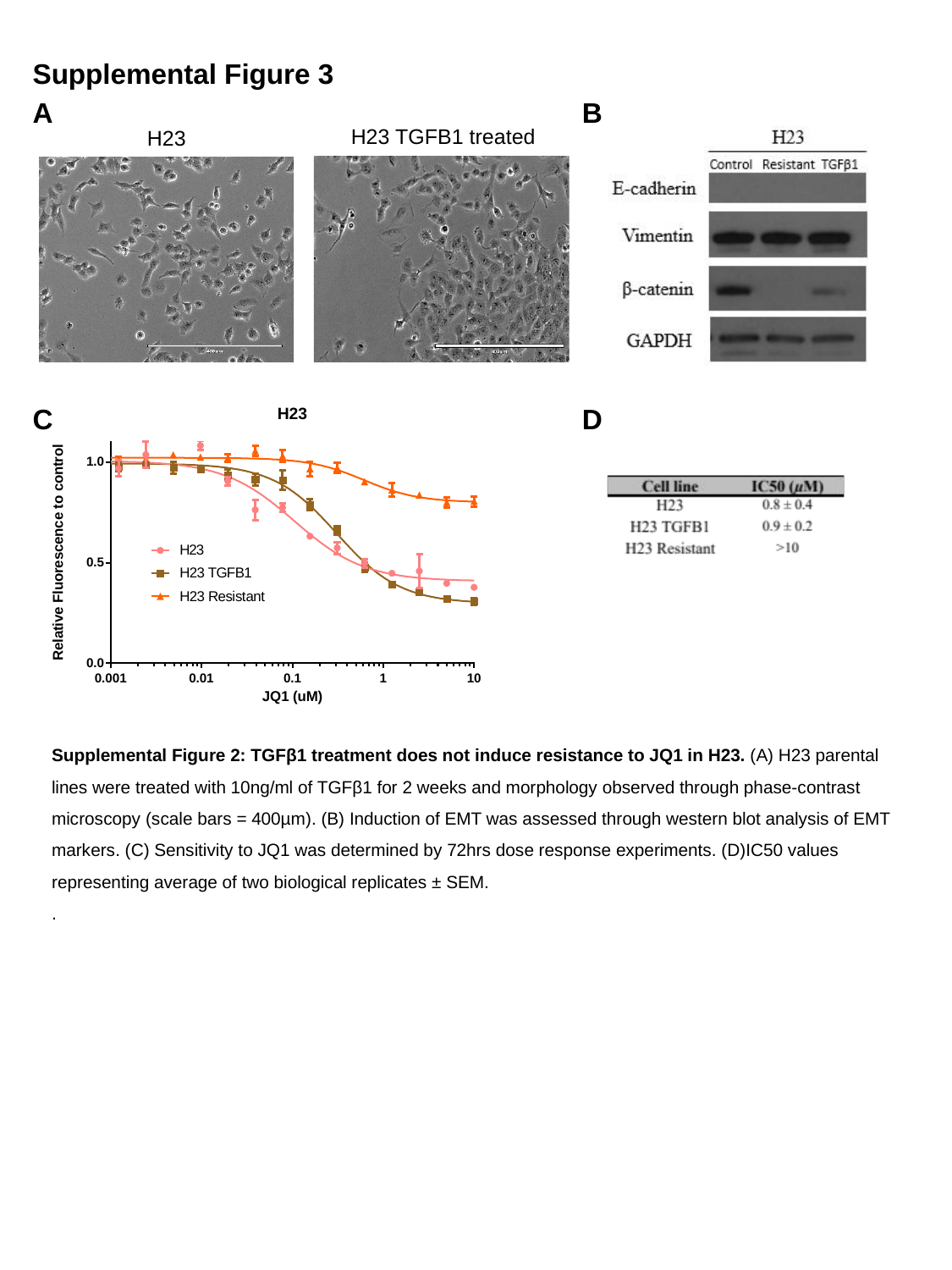

Supplemental Figure 3
A
B
H23 TGFB1 treated
H23
C
D
Supplemental Figure 2: TGFβ1 treatment does not induce resistance to JQ1 in H23. (A) H23 parental lines were treated with 10ng/ml of TGFβ1 for 2 weeks and morphology observed through phase-contrast microscopy (scale bars = 400µm). (B) Induction of EMT was assessed through western blot analysis of EMT markers. (C) Sensitivity to JQ1 was determined by 72hrs dose response experiments. (D)IC50 values representing average of two biological replicates ± SEM.
.

## Slide 4
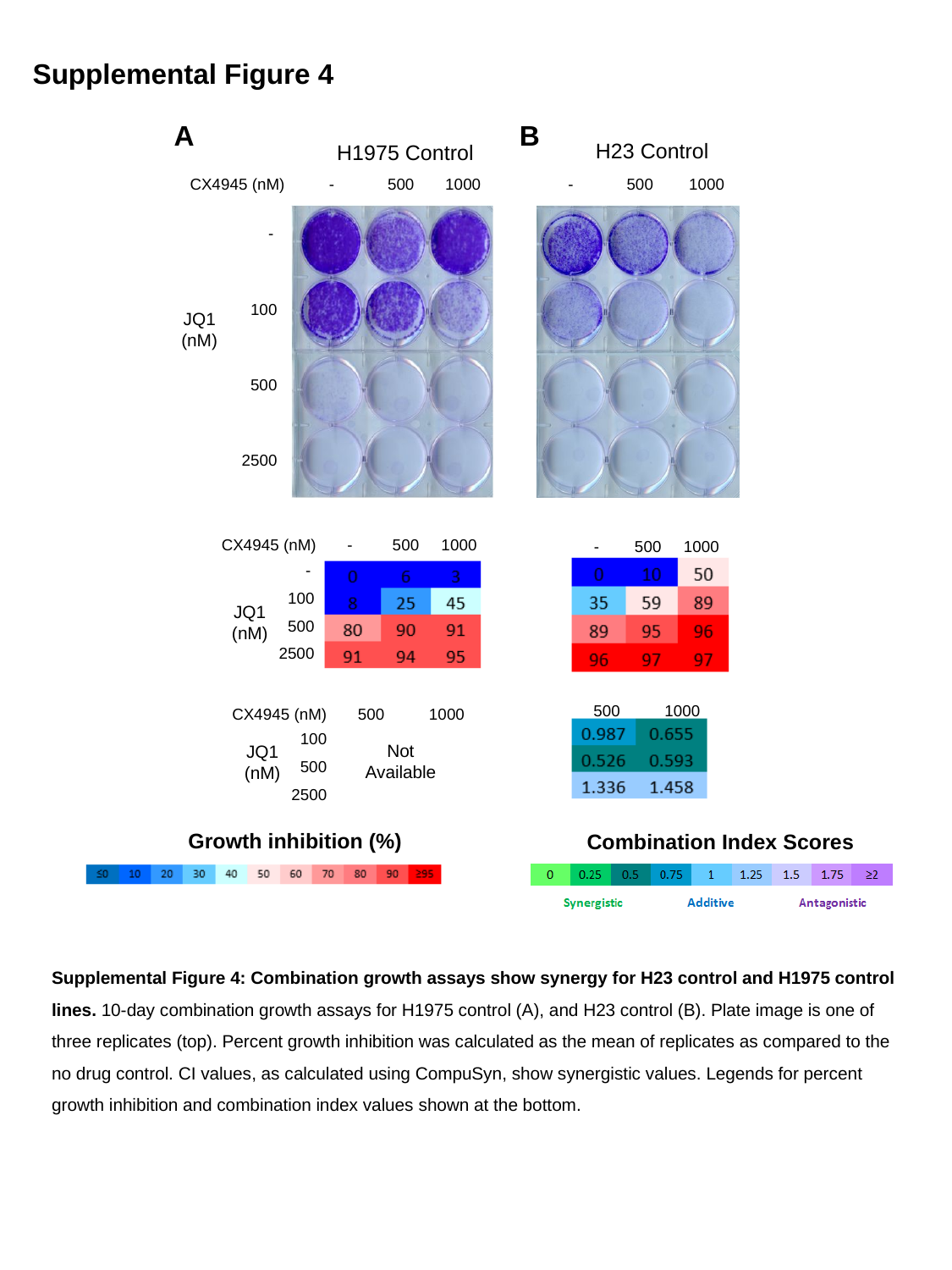

Supplemental Figure 4
A
B
H23 Control
H1975 Control
CX4945 (nM) - 500 1000
- 500 1000
 -
 100
 500
2500
JQ1
(nM)
CX4945 (nM) - 500 1000
- 500 1000
 -
 100
 500
2500
JQ1
(nM)
 100
 500
2500
500 1000
CX4945 (nM) 500 1000
Not Available
JQ1
(nM)
Growth inhibition (%)
Combination Index Scores
Supplemental Figure 4: Combination growth assays show synergy for H23 control and H1975 control lines. 10-day combination growth assays for H1975 control (A), and H23 control (B). Plate image is one of three replicates (top). Percent growth inhibition was calculated as the mean of replicates as compared to the no drug control. CI values, as calculated using CompuSyn, show synergistic values. Legends for percent growth inhibition and combination index values shown at the bottom.

## Slide 5
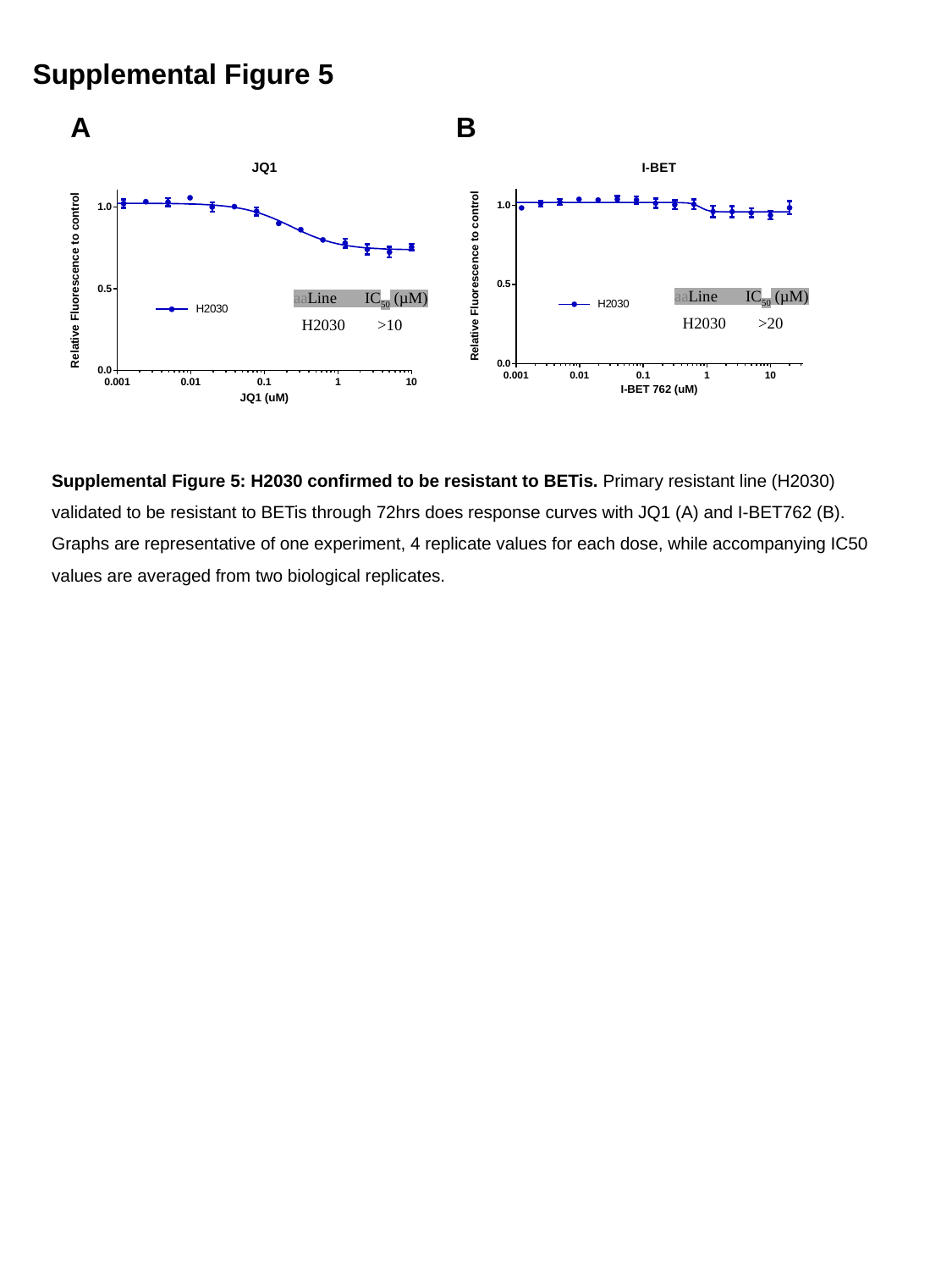

Supplemental Figure 5
A
B
aaLine IC50 (µM)
 H2030 >20
aaLine IC50 (µM)
 H2030 >10
Supplemental Figure 5: H2030 confirmed to be resistant to BETis. Primary resistant line (H2030) validated to be resistant to BETis through 72hrs does response curves with JQ1 (A) and I-BET762 (B). Graphs are representative of one experiment, 4 replicate values for each dose, while accompanying IC50 values are averaged from two biological replicates.
